# Exploring Embryonic and Postnatal Gene Therapy Approaches for GJB2-Related Deafness: A Scoping Review

**DOI:** 10.3390/audiolres16020049

**Published:** 2026-03-25

**Authors:** Valeria Caragli, Alessandro Martini

**Affiliations:** 1 Otorhinolaryngology-Head and Neck Surgery, Audiology Program, University of Modena and Reggio Emilia, 41125 Modena, Italy; 2 Padova University Research Center “International Auditory Processing Project in Venice (I-APPROVE)”, Department of Neurosciences, University of Padua, 35128 Padua, Italy

**Keywords:** GJB2 gene, hearing loss gene therapy, embryonic and postnatal intervention, ethical considerations, genetic therapy

## Abstract

*Purpose:* Hearing loss (HL) is a prevalent condition significantly impairing quality of life, with genetic mutations accounting for a substantial proportion of congenital cases, notably those involving the GJB2 gene encoding connexin 26. This study aims to analyze the current knowledge, feasibility, and challenges of gene therapy targeting GJB2-related HL, emphasizing both embryonic and postnatal interventions. *Methods:* A comprehensive scoping review was conducted across electronic databases up to October 2025, including studies focusing on GJB2-associated HL, gene therapy approaches, and the timing of interventions. Data extraction encompassed mutation types, animal models, delivery strategies, outcomes, and ethical considerations. *Results:* The results indicated over 467 GJB2 variants which could impair cochlear ion homeostasis and development. Animal models, mainly murine, demonstrated early-onset degeneration with limited recovery following delayed gene therapy, while early postnatal intervention showed greater efficacy. Viral vectors like AAV have been employed for targeted gene delivery via cochlear injections, achieving partial restoration of connexin expression and cochlear function, yet they have faced limitations including transduction efficiency, immune responses, and long-term stability. Challenges in translating these findings to humans have been compounded by anatomical, immunological, ethical, and safety issues, particularly regarding embryonic gene therapy and germline modifications. Ethical frameworks can vary internationally, highlighting the necessity for careful regulation. *Conclusions:* While promising advances in gene therapy for GJB2-related HL have been achieved in preclinical studies, significant scientific, technical, and ethical barriers must be addressed before clinical application, especially during embryogenesis. A multidisciplinary, cautious approach is essential to realize the potential of gene therapy in restoring natural hearing while safeguarding individual and societal interests.

## 1. Introduction

Hearing loss (HL) is one of the significant factors affecting the quality of human life and causing lifelong disability. According to the World Health Organization (WHO), more than 430 million people worldwide currently live with disabling hearing loss, and this number is expected to rise to over 700 million by 2050. Congenital hearing loss occurs in approximately 1–3 per 1000 live births, and genetic factors account for nearly 50–70% of cases, making hereditary hearing loss a major contributor to pediatric auditory impairment [1].

Connexin 26 gene (GJB2) mutations are considered consider to be the cause of the majority of cases with inherited, non-syndromic sensorineural HL [2], often with a recessive inheritance of a mutant gene or a dominant-negative mutation of the gene itself [2]. The GJB2 gene was first identified by Kelsell et al. in 1997 [3] as a causative gene for SNHL. Among connexin genes, *GJB2* (encoding Cx26) and *GJB6* (encoding Cx30) are the predominant connexin genes and are widely expressed, following the embryological stages (P0-P3 in mice), in the supporting cells (SCs) of the cochlear epithelium and the connective tissues of the inner ear [4]. Here, they are responsible for intercellular substance transfer and signal communication, sustaining the spontaneous electrical activity from hair cells and maintaining intracellular and extracellular K^+^ cycling, Ca^2+^ waves, cell communication, and the exchange of nutrients. Connexins play a crucial role in cochlear physiology, particularly through their involvement in periodic ATP production. This ATP release promotes glutamate secretion, which in turn triggers action potentials in spiral ganglion neurons (SGNs). The activation of SGNs is essential for proper auditory signaling and for the maturation of the cochlea, enabling the transition from Kolliker’s organ to a fully functional organ of Corti [4]. From a molecular–genetic perspective, more than 467 different pathogenic variants have been identified in connexin genes, including missense and nonsense mutations, frameshifts, insertions, and deletions [2]. Among these, the most prevalent GJB2 mutations are 35delG in European populations, 167delT in Ashkenazi Jewish individuals, and 235delC in East Asian populations. All three variants result in a functional null allele of Cx26 and are therefore expected to produce phenotypic effects comparable to those observed in functional null mouse models [5,6]. In addition to Cx26, pathogenic mutations affecting other connexins—such as Cx30, Cx31, and Cx29—have also been reported as causes of hereditary hearing loss. In rehabilitating auditory function due to GJB2 mutations, hearing aids are only suitable for hearing rehabilitation for patients with moderate to severe HL nowadays, while cochlear implants are the gold-standard auditory treatment in patients with GJB2-related severe to profound hearing loss [7]. However, sounds still remain muffled by CI to one degree or another, as band pass filtering and/or spectral smearing provide a close but not fully natural quality of hearing.

Thus, in order to restore hearing with more natural characteristics of sounds, recent studies have explored different approaches, such as gene therapy and hair cell (HC) regeneration [8], supported by technological progresses, to bring these possibilities closer to an effective solution for hearing loss [9].

The world’s first clinical trials targeting the *OTOF* gene for inherited hearing loss took place in both China and the UK, with the results showing restored hearing in children. In these trials, a working copy of the *OTOF* gene was delivered to the inner ear using a harmless virus via a surgical procedure similar to cochlear implant surgery, demonstrating promising therapeutic outcomes for patients with OTOF-related HL [10,11,12]. Similarly, due to the prevalence and crucial role of connexins. both in embryological and postnatal stages, researchers are now focusing on GJB2 gene therapy; however, more difficulties in technical management and ethical issues have emerged in this regard.

The aim of this study is to perform an analysis of current knowledge, feasibility, and challenges regarding gene therapy for GJB2-related hearing loss, in both the embryonic period and the postnatal stage, and to evaluate the potential advantages and disadvantages of such a treatment, with particular emphasis on the embryonic era.

## 2. Material and Methods

A scoping review through different electronic databases, including PubMed, Embase, and Scopus, was performed, covering publications up to October 2025 and without any time filtering in order to evaluate (1) the scientific and clinical feasibility of *GJB2*-targeted gene therapy; (2) the potential benefits and limitations of such therapy when applied during embryonic development versus after birth; and (3) the methodological, technical, and ethical challenges associated with implementing gene therapy for hearing loss. The search included a combination of MeSH terms and keywords, such as (1) (GJB2 OR connexin 26) AND (hearing loss)) and (2) ((GJB2 OR connexin 26) AND (hearing loss) AND (gene therapy OR genetic treatment)). Non-English studies were excluded. Reference lists of the relevant studies were also hand-searched for additional sources.

Studies were included if they met the following criteria: (1) focused on *GJB2*-related hearing loss; (2) described or evaluated gene therapy approaches (in vitro, in vivo, or clinical); (3) addressed the embryonic or postnatal timing of gene therapy; and (4) reported on outcomes, feasibility, or challenges related to gene therapy. Studies were excluded if they (1) focused solely on non-genetic causes of hearing loss; (2) did not include any experimental or review data on gene therapy; (3) were purely theoretical without references to empirical findings; and (4) were editorial or opinion pieces without original analysis or data.

Title and abstract screening were independently conducted by two reviewers (VC, AM). The full texts of potentially eligible studies were assessed for inclusion. Data extraction was performed independently by reviewers to ensure accuracy and comprehensiveness. The information extracted included the study design, gene mutation type, gene technology used, research models, audiology application, measured outcomes, and key findings. Qualitative results synthesis was performed.

Data extraction focused on several parameters reported in the selected studies, including study design, mutation type, delivery technology, experimental model, and measured outcomes. Particular attention was given to objective functional and molecular indicators of therapeutic efficacy, such as auditory brainstem response (ABR) thresholds, distortion product otoacoustic emissions (DPOAE), and connexin 26 protein expression levels assessed through histological or molecular techniques.

These parameters were used in the reviewed experimental studies to evaluate the impact of gene therapy on cochlear function and structure. When available, functional hearing outcomes, particularly ABR threshold improvements, were considered the primary indicators of auditory recovery following gene delivery. These objective outcomes also allowed indirect comparison of the effects of embryonic versus early postnatal interventions, highlighting the importance of timing for therapeutic success.

## 3. Results

**Figure 1:**
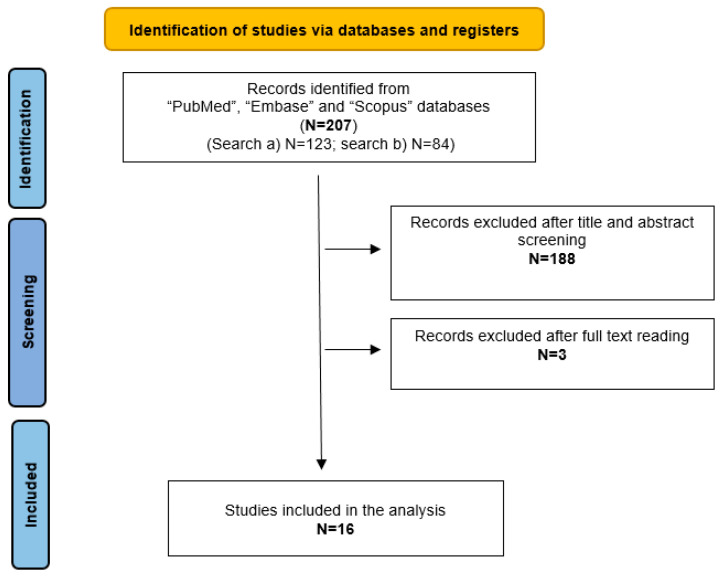
Study selection process.

A total of 123 and 74 studies were retrieved from search (1) and search (2), respectively. After title and abstract screening, 178 articles were excluded because they were off topic or duplicates, while 19 articles were selected for full-text reading. Finally, a total of 16 studies were considered and ultimately included in the scoping review as they met the search criteria. Figure 1 summarizes the article selection process.

The studies included in this review were categorized according to the study design and experimental model in order to facilitate interpretation of the data. The majority of studies consisted of in vivo experimental investigations using animal models, primarily genetically modified mice, while a smaller number involved in vitro cellular or organotypic models. Additionally, several studies were reviews or translational analyses discussing emerging therapeutic strategies and clinical perspectives.

Globally, the included studies encompass a broad range of experimental designs, reflecting the heterogeneity of research approaches used to investigate GJB2-related HL and its potential therapeutic modulation. The settings were largely preclinical and experimental, with small and highly controlled populations, primarily composed of transgenic mouse models. Human-based studies were few and descriptive, focusing on clinical characterization rather than therapeutic interventions. Table 1 details the studies’ designs, intervention types, and main outcomes.

### 3.1. Mutation Types and Frequency

Analyzing the type and frequency of gene mutations, we noticed that numerous recessive mutations in GJB2 (encoding connexin 26, Cx26) have been identified, totaling over 90 variants, including nonsense, missense, splicing, frameshift mutations, and in-frame deletions [13]. Specifically, the most prevalent mutations were deletions, such as 35delG and 235delC, which can result in the loss of gene function. Additionally, GJB6 mutations such as GJB6-D13S1830, which impacts connexin 30 (Cx30), were common [13].

These mutations impaired gap junction (GJ) channel formation, disrupting ionic homeostasis, particularly potassium recycling, calcium signaling, and metabolic support, within the cochlea [9]. The loss of Cx26 or Cx30 can lead to defective intercellular communication, abnormal connexin trafficking, and disrupted cochlear development, notably impairing the opening of the tunnel of Corti and the maturation of the organ of Corti [4]. Haploinsufficiency or dominant-negative effects can further exacerbate dysfunction, resulting in congenital sensorineural hearing loss [14].

**Table 1:** Design, intervention type and main outcome of the included studies.

Ref.	Study Design	Model/Population	Key Methods	Intervention Type	Main Outcome
[2]	Experimental in vivo	Dominant-negative mouse model	AAV-ABE editing	Gene editing	Restoration of gap junctions
[8]	Experimental in vivo	Conditional GJB2 null mice	AAV + dexamethasone	Gene + drug therapy	Enhanced rescue effect
[9]	Review/experimental synthesis	Mouse & zebrafish models	Molecular analysis	Conceptual	Therapeutic perspectives
[14]	Experimental in vivo	Cx30 knockout mice	Transgenic expression	Genetic rescue	Hearing restored by Cx26 expression
[15]	Experimental in vivo	Cx26 null mice	BDNF therapy	Neuroprotection	Neuronal survival
[16]	Experimental animal study	Conditional GJB2 KO mice	AAV-GJB2 transfer	Gene therapy	Limited functional rescue
[17]	Experimental in vivo	Mouse model	AAV-Cx26 injection	Gene therapy	Partial hearing rescue
[18]	Experimental in vivo	Mice	Liposome gene delivery	Gene transfer	Transient hearing impairment
[19]	Experimental mechanistic	Cellular models	siRNA technology	Gene suppression	Proof of concept
[20]	Experimental preclinical study	Mouse model	Microsurgical cochleostomy, scala media access, inner ear injection techniques, histological assessment	Surgical approach for gene delivery	Establish a reproducible surgical technique
[21]	Experimental in vivo	Cx30 null mice	Scala media injection	Gene therapy	Transient hearing preservation
[22]	Narrative review (preclinical and clinical studies)	Preclinical animal models and human clinical trials	Gene replacement, gene editing, and RNA-based therapies	Gene replacement, gene editing (CRISPR), RNA-based therapies	Highlight significant translational progress
[23]	Experimental in vitro	Cochlear organotypic cultures	BAAV gene transfer	Gene therapy	Recovery of intercellular coupling
[24]	Experimental transplantation	Guinea pig cochlea	Amniotic cells	Cell transplantation	Expression of Cx26
[25]	Review article	Embryonic and fetal models (preclinical) and emerging clinical applications	CRISPR/Cas systems, delivery platforms, off-target analysis, ethical considerations	Genome editing (CRISPR/Cas-based)	Identify key barriers
[26]	Experimental preclinical study	DFNB1 mouse models (GJB2-related hearing loss)	AAV vector design, cochlear injection, auditory brainstem response (ABR), histology	AAV-mediated gene replacement therapy	Demonstrate improvement of hearing function and cochlear cellular phenotype

### 3.2. Animal Models of GJB2-Related Deafness

Focusing on animal models used to study GJB2-related deafness and related gene therapy, it emerged that mouse models with targeted Gjb2 deletions or mutations, including conditional knockouts (CKO), knock-ins (e.g., 35delG), and CRISPR-induced deletions, were extensively employed [9,15,16]. These were largely used due to the rapid onset of deafness, cochlear structural abnormalities, and cellular degeneration, particularly in the hair cells and supporting cells.

In this regard, Takada [15] performed a deletion of Cx26, causing a rapid degeneration of the organ of Corti and spiral ganglion neurons (SGNs), especially in basal turns, within months. Moreover, he also noticed that Brain-derived neurotrophic factor (BDNF) provided neuroprotection but did not rescue sensory epithelium [15].

Similarly, Iizuka [17] used an AAV-mediated GJB2 delivery to restore Cx26 expression in neonatal mice (P0), although improved hearing in adults with advanced hearing degeneration was not achieved.

When using conditional knockouts, postnatal deletion led to progressive degeneration, with a therapeutic window within the first months. In this case, the timing of gene delivery was critical as early interventions (P1–P14) yielded better outcomes, while delayed therapy resulted in irreversible cell loss [9].

Moreover, CRISPR/Cas9 models were generated to replicate common deletions such as Dfnb1em274, resulting in cochlear malformation and persistent deafness [9].

Experimental research on other animals has been performed much rarer: zebrafish models were constructed via gene knockdown and transgenic approaches targeting Cx30.3 (homologous to human GJB2/Cx26), showing the developmental impact on hair cell (HC) maturation and behavioral responses with partial functional rescue, but without quantified hearing thresholds [9].

Regardless of the animal model used, the overall outcomes included partial or no functional hearing recovery depending on the intervention timing, suggesting that early gene therapy delayed degeneration, while long-term restoration has remained challenging.

### 3.3. Delivery Methods

Gene therapy approaches utilized viral vectors, such as adeno-associated virus (AAV), adenoviral vectors, and lentiviruses (LV) for transgene delivery.

Specifically, delivery methods included:

− Local microinjection via the round window membrane (RWM) or cochleostomy [18,19];
− Diffusion-based methods with liposome complexes on the RWM [20];
− Canalostomy and endolymphatic sac injections targeting specific cochlear regions;
− Viral vectors such as AAV2/1, AAV2.7m8, and BAAV employed for gene transfer, with strategies including gene replacement, base editing [2], and RNA interference [19].

The delivery timing varied, with early postnatal interventions (P1–P14) showing greater efficacy [16,17].

### 3.4. Outcomes and Limitations

GJB2 gene therapy outcomes showed partial restoration of connexin expression, improved ionic homeostasis, and, in some cases, hearing recovery [2,21,22]. For example, AAV-mediated GJB2 gene delivery in neonatal mice improved cochlear structure and function, although long-term efficacy has remained uncertain due to immune responses, limited transduction efficiency, and cellular degeneration [16,18].

Conversely, limitations included insufficient transduction volumes and cellular specificity, particularly in mature cochlea [23], potential immune reactions, and epithelial damage caused by the injection procedures. Moreover, challenges in achieving stable, long-lasting expression and functional integration, as well as the differences between animal models and human pathology, are still present [9].

### 3.5. Human Data and Translational Challenges

Gene therapy trials on humans have presented limited data while reporting preclinical stages data.

Specifically, studies on humans have confirmed that null and severe GJB2 mutations caused extensive hair cell degeneration and cochlear structural abnormalities [24]; thus, early diagnosis and intervention were crucial.

While the current preclinical evidence supported the safety and efficacy of gene therapy in postnatal stages, the development of interventions during embryogenesis has remained fraught and experimental approaches were challenging, as these concern ethical issues with the use of human embryos as well as technical methods such as delivery efficiency, immune responses, long-term safety, and differences in cochlear development compared to animal models [9].

Specifically, concerning the temporal constraints for functional recovery, the opportunity window for restoring auditory function has been limited; therefore, beyond a certain developmental stage, degeneration can become irreversible and therapeutic interventions can exhibit diminished efficacy [16].

A significant toxicity risk of embryonic gene therapy was also linked to the possible ectopic expression of the inserted gene—where the transgene is expressed in non-target cells, such as cochlear hair cells [25]. Studies emphasized the necessity of employing cell-specific promoters to mitigate off-target effects and prevent cellular damage [26]. Achieving efficient and specific delivery of vectors (AAV or alternative vectors) to target cochlear cells has remained a major barrier [27]. Moreover, translating successful outcomes on mice to human embryonic tissues presented challenges such as the blood-labyrinth barrier, vector volume limitations, dosage optimization, and safety.

Similarly, genome editing technologies (CRISPR/Cas9, TALENs) carried risks of off-target modifications, insertions, deletions, and mosaicism—where only a subset of cells is corrected—which can be particularly problematic in the context of embryonic editing [28].

Ethical debates emphasized that unintended genetic alterations could have deleterious consequences, and mosaicism could complicate achieving uniform gene correction across all the relevant cells.

Ensuring sustained gene expression without epigenetic silencing, toxicity, or interference with cellular processes was critical too. The variability of phenotypic expression based on the mutation type and organismal context further complicated predicting outcomes [16].

In this regard, considering germline and multi-generational risks, genomic modifications in germline cells or embryos were heritable, raising ethical concerns about unintended transmission of errors or off-target effects to subsequent generations [29]. The potential for misuse or “enhancement” applications exacerbated ethical debates, emphasizing the need for strict oversight.

**Figure 2:**
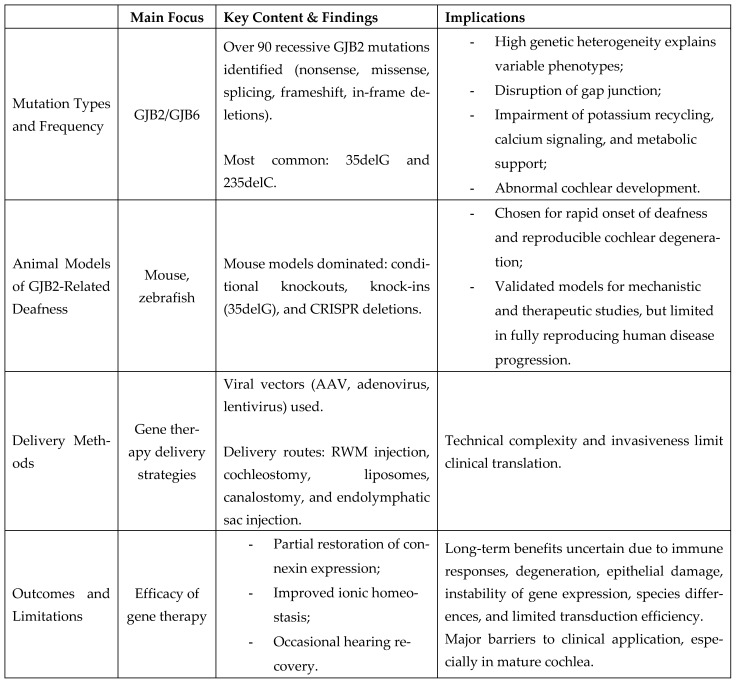
Summary of the key findings of GJB2 therapy.

Figure 2 summarizes the key concept of GJB2 therapy characteristics and its implications.

#### Ethical and Legal Considerations in Embryonic Interventions

The application of gene therapy during embryonic development significantly varied across countries, raising profound ethical, legal, and social issues.

In Italy, the legislative framework has been highly restrictive. Law 40/2004, along with subsequent amendments, has rigorously regulated research involving human embryos, limiting the scope of permissible experiments and prohibiting procedures aimed at germline modification for reproductive purposes (Law 40/2004). The Italian stance is aligned with broader European Union (EU) policies, which have generally prohibited germline modifications intended for reproduction, emphasizing the respect for human dignity, the integrity of the human genome, and the precautionary principle [30].

Conversely, the United Kingdom has permitted research on human embryos up to 14 days under strict oversight, but germline modification for reproductive use has remained prohibited [31]. Similarly, in the United States, federal policies have not explicitly banned germline editing, but they have imposed de facto restrictions through funding limitations and ethical guidelines, with individual states enacting their own regulations. China has conducted controversial experiments involving human embryo editing, prompting international criticism and calls for stringent governance [32]. Countries such as Canada and Australia have maintained strict prohibitions against germline modifications for reproductive purposes.

The core ethical issues have revolved around the absence of consent from future individuals, the potential unintended consequences, and the risk of eugenics or enhancement beyond therapeutic aims. Intervening during embryogenesis could alter the individual’s genetic identity, raising questions about the right to genetic integrity and the morality of making irreversible changes without the consent of the future person [33]. Moreover, the societal implications have included potential exacerbation of inequalities, access disparities, and the possibility of non-therapeutic “enhancement” applications, which have further complicated the ethical landscape.

**Figure 3:**
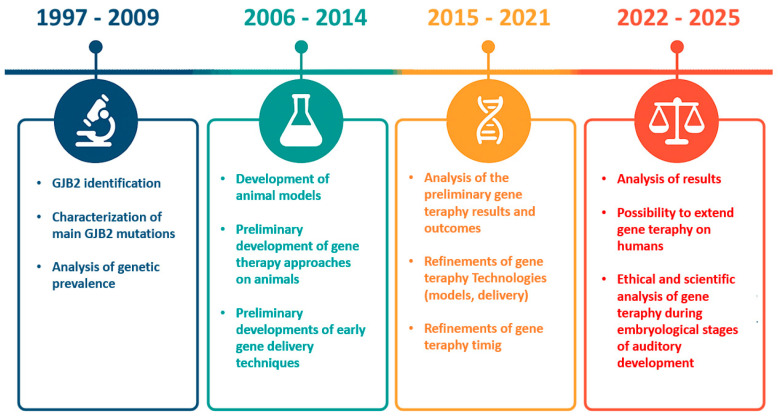
Timeline of GJB2 gene therapy development.

Figure 3 summarizes the most important advancements in GJB2 therapy over time.

## 4. Discussion

This review aimed to evaluate the current evidence regarding embryonic and postnatal gene therapy approaches for GJB2-associated hearing loss, focusing on experimental efficacy, delivery strategies, biological limitations, and translational challenges.

The pursuit of gene therapy to recover from genetic hearing loss, particularly GJB2-related deafness, has presented a promising yet complex frontier in auditory biomedical research.

**Figure 4:**
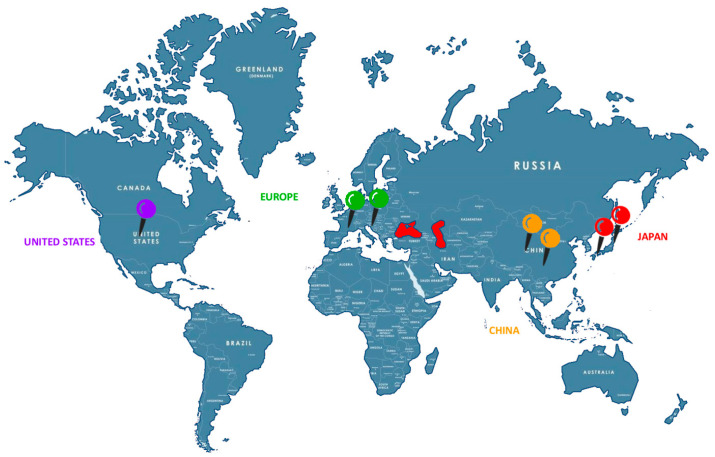
World map of active group researchers on GJB2.

From mutation identification and gene identification (1997) [3], many advances have been made since the development of valid animal models and effective early gene therapy approaches. Active countries on this topic have mostly involved Japan, followed by China, the United States, the UK, and broader Europe, with Italy primarily involved in legal/regulatory discussions rather than experimental studies (Figure 4).

Nonetheless, the current landscape has underscored significant scientific, technical, and ethical challenges that must be meticulously addressed before clinical translation can be realized. The reviewed data delineated the multifaceted nature of these obstacles, encompassing vector delivery limitations, timing of interventions, safety considerations, and ethical implications, especially those concerning embryonic treatment.

### 4.1. Mutation Spectrum and Pathophysiology

The genetic landscape of GJB2-related hearing loss is one of the first challenges that must be considered, as over 467 different mutations has been identified, predominantly affecting connexin 26 (Cx26) and connexin 30 (Cx30) [2]. This would suggest that gene therapy requires different approaches when being performed; for instance, while recessive inheritance of a mutant *GJB2* might be cured by a functional replacement with wild-type *GJB2*, for hearing loss caused by a dominant-negative mutation in *GJB2*, genome editing to restore normal gene function would be considered the best approach for treatment [2]. The temporal progression of hearing degeneration has also been a critical point for gene delivery timing [9,15]. For these reasons, gene therapies would not only require a comprehensive genetic evaluation, but also a narrow therapeutic window to be performed, emphasizing the importance of early intervention to yield better outcomes [17]. Translating these findings from animal models to human clinical practice has evidently complicated the implementation of the therapy itself.

### 4.2. Gene Therapy Approaches and Delivery Methods

Current gene therapy strategies have predominantly employed viral vectors, such as AAV, and delivery routes, including cochleostomy, round window membrane injection, and canalostomy [18,20]. The efficacy of these methods has hinged on achieving sufficient transduction efficiency, cell-specific targeting, and sustained expression, the realization of which has remained challenging. Recent advances, such as the development of novel AAV serotypes (e.g., AAV2.7m8), have shown promise in enhancing cochlear transduction and in reducing immunological damage on tissues using steroids (Dexamethasone) [8]; however, limitations have persisted, notably in mature cochlea where cellular barriers can impede vector access [23]. These findings can underscore the necessity of optimizing delivery techniques, vector design, and timing, yet they have also exposed the gaps between animal models and human application, where anatomical and immunological barriers pose additional hurdles.

### 4.3. Outcomes, Limitations, and Translational Challenges

Although the numerous practices and clinical challenges, preclinical studies, including those on humans, have demonstrated that gene therapy may both restore connexin expression and normalize ionic homeostasis, as well as partially recovering cochlear function [2,21]. As hearing impairments are detectable at or shortly after birth and the outcome of auditory treatment has been more effective when it performed as early as possible, early genetic screening could be mandatory. Nonetheless, the actual durability of current gene therapy effects has remained uncertain, with issues such as transient transgene expression, immune responses, and cellular degeneration limiting long-term efficacy. Human data have been sparse too, with ongoing trials primarily in preclinical stages, reflecting the translational gap [33]. Embryonic application has introduced additional complications, including technical delivery difficulties and profound ethical concerns, notably regarding germline modifications, consent, and heritability [33]. The risk of off-target effects, mosaicism, and unintended genetic alterations have further complicated embryonic interventions, necessitating rigorous safety evaluations and regulatory oversight.

### 4.4. Embryonic vs. Postnatal Feasibility and Comparative Effectiveness

A critical aspect emerging from the analyzed studies has been the distinction between embryonic and postnatal feasibility of gene therapy interventions for GJB2-related hearing loss. Experimental evidence consistently suggested that earlier interventions provided greater therapeutic benefit, mainly because cochlear degeneration associated with connexin deficiency began during early developmental stages [9,16]. Embryonic intervention theoretically offered the advantage of correcting the genetic defect before irreversible structural damage occurred, potentially allowing for normal cochlear maturation. However, embryonic gene therapy has raised substantial technical and ethical challenges, including safe vector delivery, potential off-target genomic effects, mosaicism, and the ethical implications of germline modification. Conversely, postnatal gene therapy, particularly during the early neonatal period, has currently appeared more feasible from both a technical and ethical standpoint. Several studies using AAV-mediated gene delivery demonstrated partial restoration of connexin expression and measurable improvements in auditory brainstem response (ABR) thresholds when treatment was administered during early postnatal stages [17,21]. Although the functional recovery was often incomplete, these findings could indicate that early postnatal intervention may represent a realistic therapeutic window in which cochlea structures can remain sufficiently preserved to benefit from gene restoration.

### 4.5. Clinical Readiness and Future Research Directions

Despite encouraging preclinical results, clinical translation of GJB2 gene therapy has remained in the early developmental stage [9,22]. The currently available evidence has been largely derived from animal models, and human data has remained limited. Several key challenges must therefore be addressed before clinical implementation can be considered. These include improving vector efficiency and specificity for cochlear-supporting cells, ensuring long-term stability of transgene expression, minimizing immune responses, and optimizing surgical delivery techniques to safely access the inner ear [23,25]. In addition, further research is needed to determine the optimal therapeutic window, define standardized functional outcome measures, and better understand genotype–phenotype correlations that may influence treatment response. Future investigations should also explore combined therapeutic strategies, such as gene therapy together with neuroprotective or regenerative approaches, to enhance auditory recovery [29]. Overall, while the current evidence has supported the promising potential of gene therapy for GJB2-related hearing loss, carefully designed translational studies and early-phase clinical trials will be essential to determine its safety, efficacy, and long-term clinical applicability.

### 4.6. Ethical and Regulatory Considerations

The ethical discourse surrounding embryonic gene therapy has been particularly contentious. The absence of consent from future individuals, the potential impacts on personal identity, and the societal implications, such as equity and risks of non-therapeutic enhancements, are critical issues [32,33,34,35,36]. The necessity of establishing international consensus, transparency, and rigorous oversight will be paramount to prevent misuse and ensure ethical integrity.

## 5. Strengths and Limitations

This review integrates evidence from multiple experimental platforms, including in vitro models, in vivo transgenic animals, and the limited human studies available, providing a broad overview of the current knowledge on GJB2-related HL and the emerging therapeutic strategies. The inclusion of recent advances in viral gene transfer and genome-editing approaches have allowed a critical appraisal of translational potential and highlighted future research directions; however, several limitations must be acknowledged. The body of evidence is predominantly preclinical, relying largely on small, highly controlled animal studies, which could limit direct applicability to human disease. Moreover, the heterogeneity of experimental designs, outcome measures, and therapeutic protocols reduces comparability across studies. Finally, the scarcity of clinical trials and long-term safety data restricts the ability to draw definitive conclusions regarding efficacy in patients, underscoring the need for well-designed human investigations.

## 6. Conclusions

Considerable progress has been made in understanding the genetic basis of GJB2-related hearing loss and the potential of gene therapy to restore auditory function. Due to the prevalence of GJB2-related HL, the pivotal role of connexins in auditory functioning since embryological stages, the severity of HL, and in light of the new technological advances, GJB2 therapy represents a fascinating target. Nevertheless, significant scientific, technical, and ethical barriers remain. Moving forward, a cautious and multidisciplinary approach—integrating advanced vector technologies, precise developmental timing, comprehensive safety assessments, and robust ethical frameworks—is essential to translate these promising preclinical findings into safe and equitable clinical solutions. Only through such rigor can the promise of gene therapy for hereditary deafness be ethically realized, respecting both individual rights and societal values.

## Data Availability

No new data were created or analyzed in this study. Data sharing is not applicable to this article.
